# Protective Effect of Galantamine against Doxorubicin-Induced Neurotoxicity

**DOI:** 10.3390/brainsci13060971

**Published:** 2023-06-20

**Authors:** Rawan S. Alsikhan, Maha A. Aldubayan, Ibtesam S. Almami, Ahmad H. Alhowail

**Affiliations:** 1 Department of Pharmacology and Toxicology, College of Pharmacy, Qassim University, Buraydah 51452, Saudi Arabia; 2 Department of Pharmacology and Toxicology, Unaizah College of Pharmacy, Qassim University, Unaizah 51911, Saudi Arabia; 3 Department of Biology, College of Science, Qassim University, Buraydah 51452, Saudi Arabia

**Keywords:** doxorubicin, galantamine, neuroinflammation, cognitive impairment, neuroprotective, ELISA, rats

## Abstract

Background and aims: Doxorubicin (DOX) causes cognitive impairment (chemobrain) in patients with cancer. While DOX damages the cholinergic system, few studies have focused on the protective effects of cholinergic function on chemobrain. The acetylcholinesterase inhibitor galantamine (GAL) demonstrates neuroprotective properties. We investigated the mechanisms associated with DOX-induced cognitive impairments and the potential protective role of GAL in preventing chemobrain. Main methods: Female Wistar rats were divided into control, DOX, GAL, and DOX + GAL groups. The rats in the DOX group were administered DOX (5 mg/kg intraperitoneally twice weekly for two weeks), while those in the GAL group were orally administered GAL (2.5 mg/kg) via oral gavage once daily for 15 days. The combination group (DOX + GAL) received GAL (once daily) and DOX (two times per week) concurrently. The body weights and survival rates were monitored daily. The animals were subjected to behavioral tests to assess the memory function followed by the biochemical estimation of inflammatory markers, including tumor necrosis factor-α (TNF-*α*), interleukine-1β (IL-1*β*), and interleukine-6 (IL-6) in rat brain tissue and RT-qPCR. Key findings: DOX caused a reduction in the body weight and survival rate, which was alleviated by GAL concomitant treatment with DOX (DOX + GAL). These groups had reduced body weights and survival rates. DOX-treated animals exhibited an impairment of short-term spatial working memory, manifested as a behavioral alteration in the Y-maze test, the novel object recognition (NOR) test, and the elevated plus-maze (EPM) test. Concurrent treatment with GAL (DOX + GAL) showed improved memory function, as evidenced by an increase in the number of entries and time spent in the novel arm, the time spent exploring the novel object, and the transfer latency in the Y-maze, NOR test, and EPM test, respectively. These findings were also supported by biochemical observations showing the reversal of DOX-induced changes in IL-1*β*, IL-6, and TNF-*α*, as well as their relative expression of mRNA in brain tissue following concurrent GAL treatment. Conclusion: GAL appeared to be a neuroprotective agent against neuroinflammation caused by DOX by reducing inflammatory markers in the brain.

## 1. Introduction

Cancer is a major leading cause of death around the world [1]; nearly 10 million people died from cancer in 2020 [2]. Despite the increased cancer mortality incidence, the survival rate of patients in developing countries improved due to early detection and quality treatment [3]. While chemotherapy is effective, toxicity and chemotherapy resistance are its main limitations [4]. Neurotoxicity resulting from chemotherapy can affect any neuron either directly (direct contact with the cell body and neurites) or indirectly (glial damage, inflammation, and other mechanisms) [5,6,7]. Chemobrain is defined as a persistent cognitive impairment, where patients experience difficulties in maintaining attention, memory, and processing speed during or after chemotherapy [8]. There is growing evidence that chemobrain is highly prevalent among patients with breast cancer due to either a direct effect of the cancer itself or nonspecific factors, where approximately 18% and 78% of patients with breast cancer experience dyscognition soon after starting chemotherapy, respectively [9,10].

An anthracycline chemotherapeutic drug, doxorubicin (DOX), is widely used in adjuvant therapy for patients with breast cancer. DOX exerts antitumor effects by intercalating DNA and inhibiting topoisomerase II [11,12]. DOX increases the level of free radicals, which damage cellular membranes, DNA, and proteins [13]. Despite its limited ability to cross the blood–brain barrier (BBB), DOX can cause severe neurotoxicity in the brain through direct or indirect mechanisms [12]. DOX-induced cognitive deficits in well-developed chemobrain are caused by multifactorial mechanisms including oxidative stress, inflammation, mitochondrial dysfunction, altered neurotransmitter levels, glial activation, the accumulation of the autophagic substrate p62, the activation of apoptosis, and the inhibition of neurogenesis [12]. In addition, choline acetyltransferase activity, the level of choline-containing compounds, and phospholipase D activity significantly decreased in the hippocampal region following treatment with DOX in mice [14].

Acetylcholine, a neurotransmitter produced by cholinergic neurons, is crucial for memory function [15]. Cholinergic innervation in the hippocampus originates primarily from the medial septum and the diagonal band, and is abundant in nicotinic acetylcholine receptors (nAChRs), specifically subtype 7 [16,17]. Furthermore, cholinergic signaling through the α7 nAChR induces long-term potentiation (LTP) and suppresses long-term depression, which influences synaptic plasticity [18]. In addition, glutamate also appears to induce LTP through α-amino-3-hydroxy-5-methyl-4-isoxazolepropionic acid (AMPA) and subsequently N-methyl-D-aspartate (NMDA) glutamate receptors. As part of LTP induction, both presynaptic and postsynaptic neurons must be simultaneously activated, as the postsynaptic neuron must be depolarized when glutamate is released from the presynaptic bouton to remove the Mg^2+^ block of N-methyl D-aspartate (NMDA) receptors. Depolarization and glutamate binding results in maximal calcium influx through NMDARs, which activate intracellular signaling cascades that are ultimately responsible for altered synaptic efficacy. Consequently, NMDAR-dependent LTP is an associative form of plasticity that strengthens neural connections [19]. Accordingly, synaptic plasticity loss is associated with dementia [19]. Surprisingly, DOX has been shown to affect learning and memory function in rodents via the impairment of synaptic plasticity in hippocampal neurons caused by AMPA receptor dysregulation [20]. Acetylcholinesterase (AChE) is primarily present at postsynaptic neuromuscular junctions and breaks down or hydrolyzes acetylcholine, which prevents it from spreading and activating nearby receptors [21]. AChE has different functions, which include influencing inflammation, oxidative stress, apoptosis, morphogenic, and adhesion functions, participating in β-amyloid accumulation [22]. Accordingly, AChE is crucial in neurodegenerative diseases [22].

The acetylcholinesterase inhibitor (AChEI) galantamine (GAL) is used to treat Alzheimer’s disease (AD). GAL does not cure AD but can improve learning, memory, and awareness by improving cholinergic function [23]. GAL blocks acetylcholine breakdown, promotes presynaptic neuron acetylcholine release, and modulates nAChRs, and has antioxidant function [24,25,26]. A unique drug, GAL, is an allosteric potentiator of α4β2 and presynaptic α7 nAChR [27], and facilitates presynaptic neuron acetylcholine release, rendering its dual mode of action clinically significant [27]. Furthermore, studies in both clinical and preclinical settings have revealed the neuroprotective properties of GAL, as well as its ability to improve cognitive performance. GAL inhibited plaque formation, attenuated amyloid deposition, and prevented neuroinflammation [28,29]. In addition, GAL facilitated the protection of dentate gyrus neurons against the lead-induced impairment of neuronal plasticity in rats [30]. Although DOX has low permeability through the BBB, the pro-inflammatory cytokines that it produces, such as interleukin-1β (IL-1*β*), interleukin-6 (IL-6), and tumour necrosis factor-alpha (TNF-*α*), are able to cross this barrier and cause local inflammatory responses in the brain, as well as oxidative stress in the brain, via the production of reactive oxygen species [31]. Furthermore, GAL inhibits inflammatory signaling molecules (NF-κB and p65) and cytokines (TNF-*α*, IL-1*β*, and IL-6), and also prevents astrocyte and microglia activation markers (CD11b and GFAP) in lipopolysaccharide-exposed mice [32].

Notwithstanding the higher percentage of chemobrain following DOX in breast cancer patients and the fact that DOX is among the most widely used anticancer antibiotics for breast cancer regimens, research that investigates the effect of DOX on the memory function of female rodents, as well as possible remedies, has been inadequate. In addition, the dearth of a scientific explanation of cognitive deficits following chemotherapy has hampered therapeutic outcomes in survivors of breast cancer patients, with higher mortality rates than without cognitive deficit. Therefore, it is quite interesting to investigate the effect of DOX on cognitive impairment in female rats and its possible amelioration using galantamine. Furthermore, the results of this research intend to incite an in-depth understanding of the underlying mechanisms of chemotherapy-induced cognitive impairment that provides an experimental tool for researching possible interventions that promote better quality of life in cancer survivors by protecting non-targeted organs from anticancer drug toxicity.

## 2. Materials and Methods

### 2.1. Drugs and Chemicals

DOX injection fluid (2 mg/mL) was obtained from EBEWE Pharma Ges.m.b.H. Nfg.KG, Unterach am Attersee, Austria, and galantamine hydrobromide was purchased from Sigma-Aldrich, St. Louis, MO, USA.

### 2.2. Animals

In this study, a total of forty-eight Wistar female rats (weighing 150–300 g) were procured from the animal house facility of the College of Pharmacy, Qassim University, Saudi Arabia. The animals were kept in propylene cages, maintained at 25 ± 2 °C under a 12 h light–dark cycle, and had free access to food and water. The rats were observed daily and their body weights were measured. The experiment was conducted in accordance with the guidelines of the College of Pharmacy Research Centre (no. 23-20-16) at Qassim University.

### 2.3. Experimental Groups and Treatment Schedule

**Figure 1:**
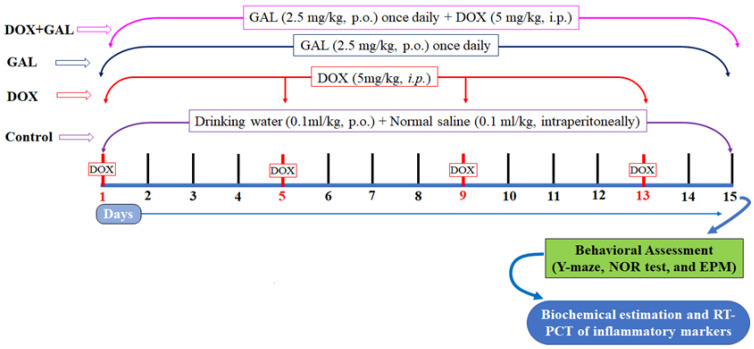
Experimental design.

The animals were randomly divided into four groups: control (n = 10), DOX (n = 18), GAL (n = 10), and DOX + GAL (n = 10). The control group was orally administered with drinking water via an oral gavage and normal saline (0.1 mL/100 g, i.p.) twice weekly for two weeks. The second group (DOX) was treated with DOX (5 mg/kg, i.p.) twice/week for two weeks (cumulative dose, 20 mg/kg) [33]. The animals in the GAL group received GAL (2.5 mg/kg) orally (p.o.) through oral gavage once a day for two weeks [34]. The DOX + GAL group administered GAL (2.5 mg/kg, p.o.) once daily and DOX (5 mg/kg, i.p.) twice weekly for two weeks. The animals were subjected to behavioral tests for an assessment of cognitive function using Y-maze, the novel object recognition (NOR) test, and the elevated plus-maze (EPM) test (Figure 1).

### 2.4. Y-maze

A Y-maze is used in rodent studies to study spatial learning and memory. The test is conducted using a Y-shaped maze with three wooden arms (measuring 50 cm × 10 cm × 18 cm, arranged at a 120° angle). Rat recognition abilities are measured by blocking off one arm of the Y-maze (novel arm). In this study, the test was performed by marking each arm as a starter arm, a familiar arm, and a novel arm. One rat was placed in the starter arm so that it could freely access the familiar arm for 10 min (training session). After 3 h, the test session was conducted with all arms open and with no restriction on maze exploration. For the test session, the animal was again placed on the starter arm and its preference for the novel arm or known arms was observed for 5 min. The test was conducted in an isolated area with good light distribution, and the apparatus was cleaned after each trial. The test sessions were video-recorded to enable an assessment of the duration that the rats spent on each arm and how many entries they made. The animal was considered to have entered an arm if all of its paws entered the arm [33].

### 2.5. Novel Object Recognition (NOR)

Various aspects of learning and memory in the rats were assessed with the NOR test. An open box made of wood (measuring 40 cm × 40 cm × 40 cm) was used with two different items (familiar objects: black cans; novel object: a small white-painted reagent bottle). Neither positive nor negative reinforcers were used in the NOR test. The rat was placed in the center of the apparatus to explore identical objects located at equal distances (training session) for 10 min. After 3 h, the test session was conducted, where a familiar object was replaced with a novel object, and the rat was exposed to it for 5 min. To avoid odor cues that could affect the animal’s behavior, the arena and objects were cleaned with 70% ethanol solution before each trial. The duration that each animal spent discovering a novel object during the test session was recorded with a video camera [33].

### 2.6. Elevated Plus-Maze (EPM) Test

The EPM test is used to evaluate memory and assess anxiety-related behaviors in rodents. A cross-shaped wooden elevated apparatus was used for this study. The apparatus consisted of two oppositely positioned closed arms (50 cm × 10 cm), two oppositely positioned open arms (50 cm × 10 cm), and a center platform (10 cm^2^). A rat was placed at the end of the open arm, facing the open area, which allowed the apparatus to be explored for 5 min. The EPM test was performed in silence, and the animal was observed via a computer-connected camera. The maze was swept after every trial. The transfer latency, the duration spent on the closed arm, and the number of entries to open and closed arms were measured with a video camera and a stopwatch. An animal was considered to have entered the arm when all of its paws were inside [33,35].

### 2.7. Brain Tissue Collection for Biochemical Analysis

The rats were placed in a glass chamber and anesthetized with CO_2_ [36], before then being killed by decapitation. The brains were removed and then their blood was washed out. Subsequently, the brains were homogenized in total protein extraction buffer with protease inhibitor, then centrifuged at 12,000 rpm for 10 min before being stored at −80 °C. The cerebellum was not collected.

### 2.8. Bicinchoninic Acid (BCA) Protein Assay

The BCA assay standard curve and protein estimation were conducted based on a previously described method [37]. A standard working reagent (200 µL) containing 100 vol BCA solution with 2 vol of copper (II) sulfate pentahydrate 4% (*w*/*v*) was added to 25.0 µL samples containing 0.1–1.2 mg of protein standard solution (bovine serum albumin). The BCA assay was performed in triplicate. The plates were incubated at 37 °C for 30 min, the absorbance was measured at 570 nm using a microplate reader, and a standard curve was plotted across a 0.1–1.2 mg/mL protein range [37].

### 2.9. Enzyme-Linked Immunosorbent Assay (ELISA)

The ELISA is a powerful analytical biochemistry technique that is used to detect and quantify specific proteins. In this study, inflammatory marker proteins in the supernatant (IL-6, IL-1*β*, and TNF-*α*) were detected with sandwich ELISA. The inflammatory marker levels were detected using rat ELISA kits for IL-1*β* (cat. no. RK00009, ABclonal Technology, Woburn, MA, USA), IL-6 (cat. no. RK00020, ABclonal Technology), and TNF-*α*1 (cat. no. RK00029, ABclonal Technology). The ELISA was performed according to the manufacturer’s instructions.

### 2.10. Reverse Transcription–Quantitative PCR (RT-qPCR)

An RT-qPCR is a sensitive and quantitative technique used to quantify mRNA expression levels. In this study, the mRNA levels of inflammatory cytokines (TNF-*α* and IL-6) were detected. The total RNA was extracted from tissue samples according to the manufacturer’s protocol using a GET Total RNA kit (cat. no. 787-123, Biosciences, San Diego, CA, USA). The oligo primers (Integrated DNA Technologies, Coralville, IA, USA) were designed using the PrimerQuest Tool, diluted to 10 µM/μL using double-distilled water, and stored at −20 °C. The RNA purity of each sample was determined using a NanoDrop ND-2000c spectrophotometer (Thermo Fisher Scientific, Labtech, UK). Integrated DNA Technologies tools were used to design a specific primer for each gene for the RT-PCR (Table 1), and 10 µM/μL working concentrations were prepared. Reverse transcription and PCR quantification were conducted using an ABScript II One-Step SYBR Green RT-qPCR Kit (cat. no. RK20404, ABclonal Technology). RNA (400 ng per sample) was reverse-transcribed into complementary DNA, and the PCR was run using an AriaMx Real-Time PCR System (Agilent Technologies, Santa Clara, CA, USA) according to the manufacturer’s instructions. A mixture of SYBR green RT-qPCR buffer, ABScript II enzyme mix, 10 μM of each forward and reverse primer, ROX II reference dye (50×), and total RNA was prepared and topped up to 20 µL with RNase-free water. The thermocycling conditions were as follows: reverse transcription consisted of one cycle for 5 min at 42 °C and pre-denaturation consisted of one cycle for 1 min at 95 °C followed by 40 reactions for 5 s at 95 °C and 32–34 s at 60 °C.

To ensure the validity of the results, the samples were run in duplicate in three independent experiments. The data were analyzed automatically using AiraMx software after setting the plate for the comparative quantitation experiment. The gene expression levels were normalized with the housekeeping gene *Gapdh*. To determine changes in the mRNA expression, transcript abundance was calculated for each gene relative to *Gapdh* transcript abundance.

### 2.11. Statistical Analysis

All results are presented as the mean ± SEM and were analyzed using GraphPad Prism 9 software (GraphPad, Boston, MA, USA). All the data (body weights; survival rates; Y-maze, NOR, and EPM tests; biochemical assay) were analyzed using a one-way analysis of variance (ANOVA), followed by Dunnett’s analysis. The data of the treatment group were compared with the data of the control group. *p* < 0.05 was considered statistically significant.

## 3. Results

### 3.1. Effect of DOX and GAL on Body Weight and Mortality

**Figure 2:**
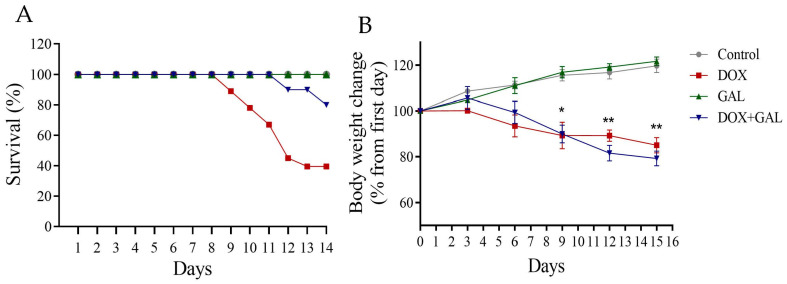
(**A**) The effect of DOX and GAL on the survival rate of rats. (**B**) The effect of DOX and GAL on changes in the body weight of rats. The data are expressed as the mean ± SEM. * *p* < 0.05, ** *p* < 0.01.

The DOX and DOX + GAL groups had reduced body weights compared with the control group. In contrast, the GAL group had increased body weight (Figure 2A). The DOX and DOX + GAL groups had higher mortality rates (Figure 2B) (DOX mortality rate:33%). No mortality was reported in the control or GAL groups.

### 3.2. Effect of DOX and GAL on Y-maze Performance

**Figure 3:**
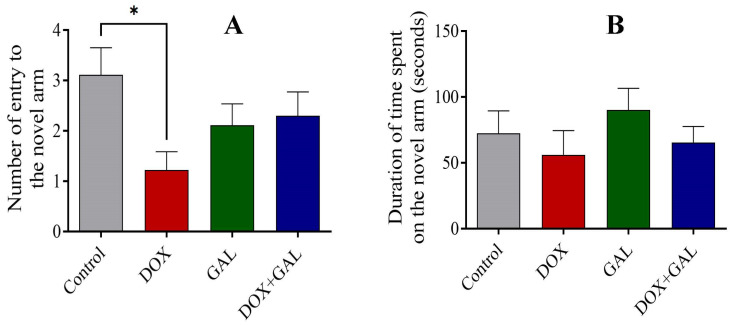
The effect of DOX and GAL on the behavior of rats on the Y-maze test. (**A**) The effect of DOX and GAL on the number entries into the novel arm. (**B**). The effect of GAL and DOX on the duration spent on the novel arm. The data are expressed as the mean ± SEM. The data were analyzed using a one-way ANOVA with Dunnett’s analysis and were considered significant when * *p* < 0.05 as compared to the control group.

The control group had a higher number of entries into the novel arm, whereas the DOX group recorded the lowest number of novel arm entries (Figure 3A), which may reflect a reference spatial memory deficit. GAL administration resulted in improved entries and duration spent in the novel arm compared to DOX treatment. There was no significant difference in the duration spent in the novel arm between the control and DOX groups (Figure 3B).

### 3.3. Effects of DOX and GAL on NOR Test Performance

**Figure 4:**
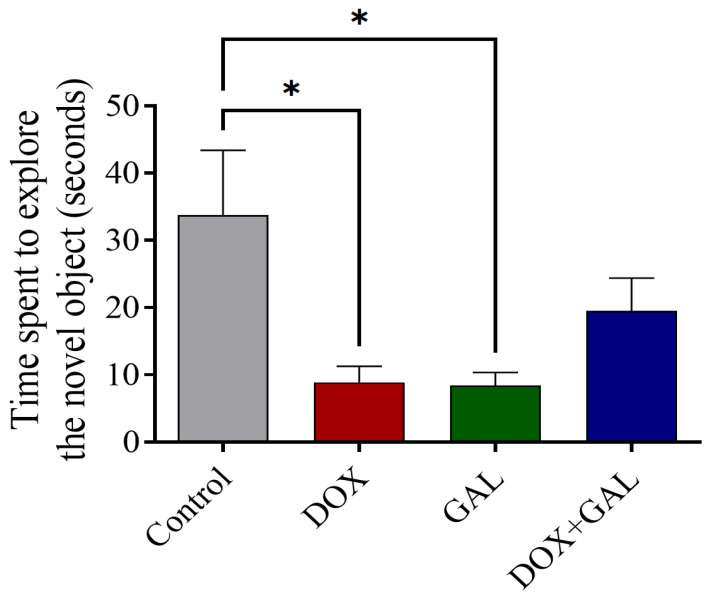
The effect of GAL on the novel object recognition (NOR) test, DOX-induced reduction, and time spent to explore the novel object(s) in rats. The data are expressed as the mean ± SEM. The data were analyzed using a one-way ANOVA with Dunnett’s analysis and were considered significant when * *p* < 0.05 as compared to the control/DOX group.

The DOX- and GAL-treated groups spent significantly less time exploring the novel object compared to the control group (Figure 4). However, GAL co-treatment prolonged the novel object exploration compared to DOX alone. These findings indicate that GAL may improve the memories of rats with DOX-induced chemobrain.

### 3.4. Effects of DOX and GAL on EPM Test Performance

**Figure 5:**
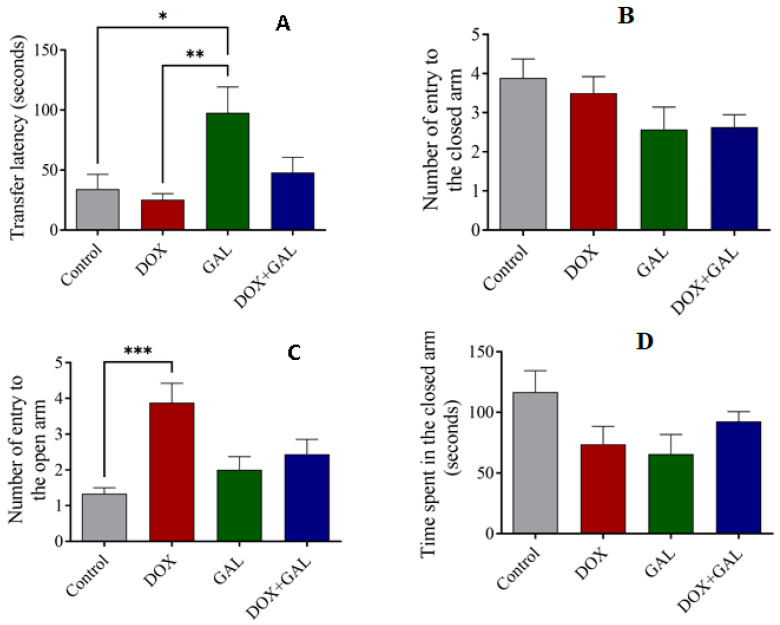
The effect GAL on DOX-induced behavioral changes in the EPM test on rats. (**A**) Transfer latency time; (**B**) the number of entries into the closed arms; (**C**) the number of entries into the open arm; and (**D**) the time spent in the closed arm of the EPM test. The data are expressed as the mean ± SEM. The data were analyzed using a one-way ANOVA with Dunnett’s analysis and were considered significant when * *p* < 0.05, ** *p* < 0.01, and *** *p* < 0.001 as compared to the control/DOX group.

The transfer latency between the GAL with DOX (*p* < 0.01) and control (*p* < 0.05) groups was significantly different. However, the total duration spent in the closed arms was not significantly different between the four groups. The DOX group had the lowest transfer latency, whereas the transfer latency was the highest in the GAL and DOX + GAL groups (Figure 5A). Furthermore, the control and DOX + GAL rats spent more time in the closed arm (Figure 5B). The DOX rats recorded the highest number of entries into the open arm, which was significantly different from the control and GAL groups (Figure 5D). There were no significant differences in the number of entries into the closed arm between the groups (Figure 5C).

### 3.5. Effects of DOX and GAL on Inflammatory Markers

**Figure 6:**
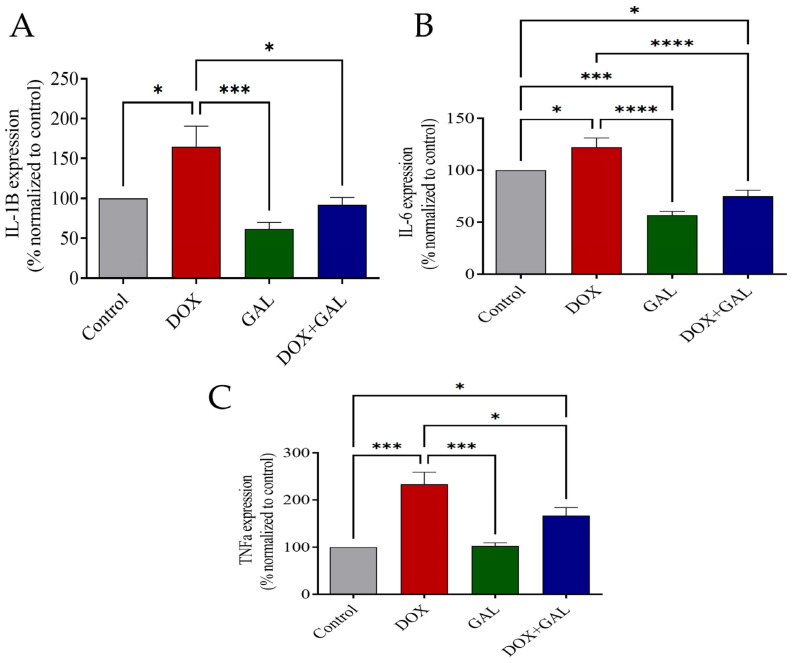
The effect of GAL on DOX-induced changes inflammatory markers. (**A**) IL-1*β*, (**B**) IL-6, and (**C**) TNF-*α* in the brain of rats. The data were analyzed using a one-way ANOVA with Dunnett’s analysis and were considered significant when * *p* < 0.05, *** *p* < 0.001, and **** *p* < 0.0001 as compared to the control/DOX group.

The DOX group had significantly increased IL-1*β*, IL-6, and TNF-*α* levels in the brain compared to the control group (Figure 6A–C). In contrast, GAL significantly diminished brain inflammation compared to DOX-injected rats. Additionally, the GAL group had significantly reduced IL-6 in comparison to the control group (Figure 6B).

### 3.6. Effects of DOX and GAL on Inflammatory Marker mRNA Expression

**Figure 7:**
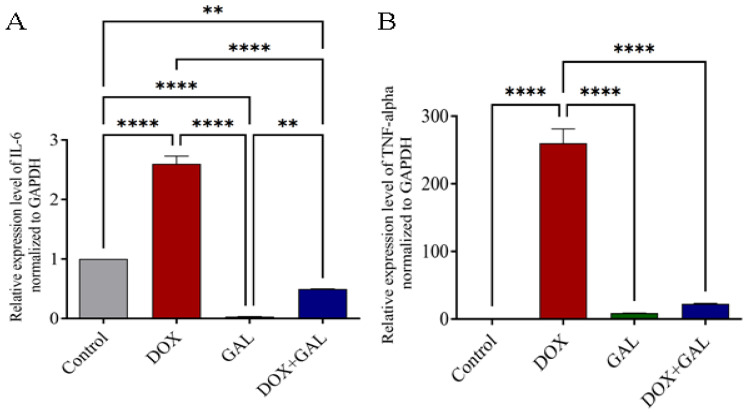
The effect of GAL on the relative mRNA expression of inflammatory markers in the brain of rats. (**A**). IL-6 expression and (**B**) TNF-*α* expression levels in the DOX treatment rat’s brain. The data were analyzed using a one-way ANOVA followed by Dunnett’s analysis and were considered significant when ** *p* < 0.01 and **** *p* < 0.0001 as compared to the control/DOX group.

IL-6 and TNF-*α* gene expression increased significantly following DOX administration (*p*< 0.0001) (Figure 7A,B), and GAL co-treatment decreased this overexpression. In comparison with the control group, the GAL group had significantly reduced IL-6 expression. This indicates that GAL had anti-inflammatory properties.

### 3.7. Collective Diagram of Results

**Figure 8:**
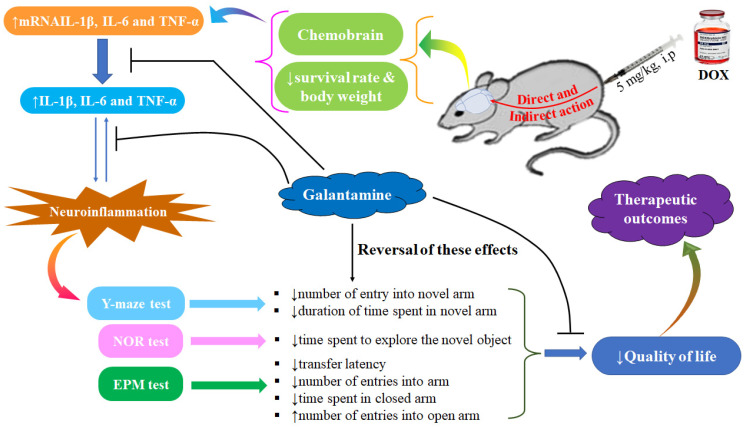
Collective diagram of the results.

Figure 8 summarizes the findings of the study, and GAL reverses the changes induced by DOX in the rats.

## 4. Discussion

Clinical oncology advances over the past few decades have significantly improved the long-term survival of patients with cancer [38]. Many cancer survivors may experience different complications lasting months or years after treatment, including cognitive impairment [38]. In this study, we hypothesized that GAL may prevent and improve DOX-induced cognitive dysfunction through its neuroprotective actions. Furthermore, DOX may cause increased AChE, which increases acetylcholine hydrolysis and contributes to inflammation [39,40]. Using GAL will compensate for acetylcholine reduction and contribute to improving memory and reducing inflammation.

In this study, DOX was used to induce a model of chemobrain. The effects of GAL were evaluated using behavioral tests such as the Y-maze, where animal performance is linked to the majority of brain regions, such as the hippocampus, basal forebrain, and prefrontal cortex [41]. The results show that DOX caused spatial memory deficiencies, evidenced by the lower number of novel arm entries. In contrast, GAL treatment improved the number of entries and the percentage of duration in the novel arm, indicating spatial memory improvement.

The rat discrimination ability was assessed with the NOR test. Among the four groups, the DOX rats demonstrated the reduced ability to discriminate between familiar and novel objects. Therefore, this paralleled previous studies that reported a significant deficit in working memory [33,39]. Nevertheless, the NOR test demonstrated that GAL co-treatment with DOX improved exploration time, indicating that GAL prevented DOX-induced cognitive deficits. The improved effects of GAL on DOX-induced cognitive impairment may be related to nAChR activation, where nAChR activation improved recognition memory in Aβ-treated animals and methamphetamine-treated mice [42,43]. Noda et al. suggested that muscarinic acetylcholine receptors (mAChRs) have little influence on the effects of GAL on specific cognitive tasks (NOR test), as scopolamine does not block the effects of GAL at the dose that impairs the performance of saline-treated mice [43].

In the EPM test, the control and DOX groups had the lowest transfer latency, with higher entries and durations in the closed arm, indicating anxiety-like behavior. The DOX group recorded more entries to the open arm, which could indicate memory impairment (amnesia) [44]. The increased transfer latency and decreased entries to the open arm in the GAL group suggested anxiety-induced behavioral inhibition, as previous studies reported that increased acetylcholine levels following the inhibition of AChE in mouse hippocampus induced anxiety-like behaviors [45].

TNF-*α*, IL-1*β*, and IL-6 were significantly increased in the DOX group compared to the control group, which accorded with previous studies in which DOX activated neuroinflammatory mediators [12]. The increased peripheral TNF-*α* levels following DOX administration disturbed the BBB, which increased TNF-*α* penetration in the brain tissues and initiated the central neuroinflammatory process via further TNF-*α* production in the brain. Moreover, TNF-*α* levels facilitated the local production of other proinflammatory mediators by activating NF-*κ*B and increasing IL-1*β* and IL-6 gene expression. This neuroinflammation may contribute to neural apoptosis and behavioral changes [12,46].

Increased IL-6 and IL-1*β* impaired spatial memory task learning and inhibited LTP in hippocampal slices [47,48,49]. Furthermore, blocking IL-6 enhanced LTP and improved long-term memory in a task that is dependent on the hippocampus [50]. The GAL treatment significantly suppressed DOX-induced and increased TNF-*α* and IL-6 mRNA expression in brain tissues compared with the DOX group, and significantly reduced IL-6 compared to the control group. The reduction in inflammatory marker levels and expression in the GAL group may have resulted from acetylcholine elevation after GAL administration, leading to the stimulus of α7 nAChR on immune cells, which caused a calcium ion influx in the immune cells. The increased calcium ion levels activated the NF-κB pathway, reducing proinflammatory cytokine production [51,52].

Inflammation is involved in psychiatric illness [53,54]. Increased peripheral or central IL-6 levels are associated with depression [55,56]. IL-6-induced depression may result from the activation of the hypothalamic–pituitary–adrenal axis or from influencing neurotransmitter metabolism, where IL-6 causes reduced serotonin, as indicated in a study which reported that IL-6 directly influenced serotonin reuptake by controlling serotonin transporter levels [56,57]. Therefore, the GAL reduction effect on IL-6 may be involved in controlling depressive symptoms in cancer survivors. These findings are consistent with previous reports which state that GAL reversed memory impairment, the inflammation of hippocampal neurons, and synaptic plasticity caused by lipopolysaccharide in mice [32].

Typically, GAL is prescribed to treat mild-to-moderate confusion (dementia) [58,59]. Using a rat model, we evaluated the beneficial effects of GAL on chemotherapy-induced cognitive deficits. The results demonstrated that GAL can be used as a therapeutic target to reduce DOX-induced cognitive deficits by enhancing cholinergic transmission, inhibiting proinflammatory cytokine release, and enhancing cognitive ability. These findings can be used to explore additional evidence to support the further clinical use of acetylcholinesterase inhibitors against chemobrain.

## 5. Conclusions

The findings of this study reveal that DOX treatment can initiate the impairment of memory function and the elevation of inflammatory cytokines and their relative expression in the brain. The administration of GAL as a co-treatment reverses these effects. Therefore, GAL appears to be a plausible candidate for treating cognitive dysfunction in the chemobrain caused by DOX treatment. In addition, GAL reduced neuronal inflammation in the brain, demonstrating a therapeutic effect.

## Figures and Tables

**Table 1:** TNF-*α* and IL-6 primer sequences used for RT-PCR.

Gene	Sequence (5′–3′)	Length (bp)
TNF-*α* FWD	ACCTTATCTACTCCCAGGTTCT	87
TNF-*α* REV	GGCTGACTTTCTCCTGGTATG	
IL-6 FWD	GCCAGAGTCATTCAGAGCAATA	87
IL-6 REV	TTAGGAGAGCATTGGAAGTTGG	
GAPDH FWD	ACTCCCATTCTTCCACCTTTG	104
GAPDH REV	CCCTGTTGCTGTAGCCATATT	

FWD: forward; REV: reverse.

## Data Availability

Available upon reasonable request.
